# Silica-coated magnetic nanoparticles impair proteasome activity and increase the formation of cytoplasmic inclusion bodies *in vitro*

**DOI:** 10.1038/srep29095

**Published:** 2016-07-05

**Authors:** Geetika Phukan, Tae Hwan Shin, Jeom Soon Shim, Man Jeong Paik, Jin-Kyu Lee, Sangdun Choi, Yong Man Kim, Seong Ho Kang, Hyung Sik Kim, Yup Kang, Soo Hwan Lee, M. Maral Mouradian, Gwang Lee

**Affiliations:** 1 Department of Physiology and Department of Biomedical Sciences, Ajou University School of Medicine, Suwon, Republic of Korea; 2 Department of Molecular Science and Technology, Ajou University, Suwon, Republic of Korea; 3 College of Pharmacy, Sunchon National University, Suncheon, Republic of Korea; 4 Department of Chemistry, Seoul National University, Seoul, Republic of Korea; 5 Pharmicell Co., Ltd. Sungnam, Republic of Korea; 6 Department of Applied Chemistry and Institute of Natural Sciences, Kyung Hee University, Yongin-si, Republic of Korea; 7 School of Pharmacy, Sungkyunkwan University, Suwon, Republic of Korea; 8 Center for Neurodegenerative and Neuroimmunologic Diseases, Department of Neurology, Rutgers–Robert Wood Johnson Medical School, Piscataway, NJ, USA

## Abstract

The potential toxicity of nanoparticles, particularly to neurons, is a major concern. In this study, we assessed the cytotoxicity of silica-coated magnetic nanoparticles containing rhodamine B isothiocyanate dye (MNPs@SiO_2_(RITC)) in HEK293 cells, SH-SY5Y cells, and rat primary cortical and dopaminergic neurons. In cells treated with 1.0 μg/μl MNPs@SiO_2_(RITC), the expression of several genes related to the proteasome pathway was altered, and proteasome activity was significantly reduced, compared with control and with 0.1 μg/μl MNPs@SiO_2_(RITC)-treated cells. Due to the reduction of proteasome activity, formation of cytoplasmic inclusions increased significantly in HEK293 cells over-expressing the α–synuclein interacting protein synphilin-1 as well as in primary cortical and dopaminergic neurons. Primary neurons, particularly dopaminergic neurons, were more vulnerable to MNPs@SiO_2_(RITC) than SH-SY5Y cells. Cellular polyamines, which are associated with protein aggregation, were significantly altered in SH-SY5Y cells treated with MNPs@SiO_2_(RITC). These findings highlight the mechanisms of neurotoxicity incurred by nanoparticles.

The use of nanoparticles (NPs) in the diagnosis and treatment of diseases has increased rapidly in recent years^1^. Magnetic nanoparticles (MNPs) and MNPs coated with biocompatible compounds are used as contrast agents in magnetic resonance imaging (MRI)-based cell labeling^2^,^3^. NPs have also enabled numerous technological advances in biomedical research. However, there are concerns regarding their toxicity and safety.

NP toxicity has typically been reported in non-neuronal cell types, while studies evaluating their toxicity to neurons are limited. There were effects of NPs reported in neurons, such as reduction of proteasome activity, decreased cell viability, increased levels of lactate dehydrogenase, triggered oxidative stress, disturbed cell cycle, induced apoptosis, and activated p53-mediated signaling pathway *in vitro*^4^. However, some types of NPs, such as silver NPs, cobalt-chromium NPs, iron oxide MNPs, and silica-coated MNPs containing rhodamine B isothiocyanate dye (MNPs@SiO_2_(RITC)), can enter the brain through endocytosis and passive diffusion without disrupting the blood brain barrier^5^. In addition, translocation of ultrafine nanoparticles to the central nervous system *via* olfactory pathway has been extensively recorded^6^. Transportation of 100, 50, and 25 nm PEGylated silica nanoparticles across the blood brain barrier (BBB) was evaluated using *in vitro* BBB and *in vivo* animal experiments^7^. Previous studies have found that certain NPs, such as *N*-iso-propylacrylamine and *N*-tert-butylacrylamide copolymer NPs, may play a role in protein fibrillization^8^. In a cellular model of Huntington’s disease, silica NPs caused nuclear protein aggregation, which was closely linked to proteasome activity^9^. Although translocation of nanoparticles into inclusion bodies inside cells has not been studied so far, there is sufficient data to suggest that these particles greatly enhance the process of protein aggregation and fibrillization^10^. Since protein aggregation precedes the formation of inclusion bodies^11^, acceleration of protein aggregation by nanoparticles can potentially contribute to neurodegenerative processes.

We have previously shown that treatment of human embryonic kidney 293 (HEK293) cells with a high concentration (1.0 μg/μl) of MNPs@SiO_2_(RITC) alters the expression of metabolic genes as well as genes related to the ubiquitin proteasome system (UPS)^12^. Falaschetti *et al*. reported modulation of the ubiquitin proteasome system by negatively charged metal oxide nanoparticles^13^. However, involvement of nanoparticles in ubiquitin proteasome dysfunction in the brain has not been investigated in depth to date. In addition, titanium dioxide nanoparticles were reported to enhance α-synuclein aggregation and reduce ubiquitin-proteasome system in dopaminergic neurons^14^. The functional status of the UPS is an important indicator of cellular homeostasis, and impaired UPS function has been linked to neurologic diseases. Alterations in the UPS can induce endoplasmic reticulum (ER) stress, which in turn can impact cellular proteasome activity and ROS generation^15^. Although ROS generation and UPS dysfunction can be countered successfully in cells as an adaptive mechanism^16^, abnormal protein aggregation and the subsequent reduction in proteasome activity are common features of neurodegenerative disorders.

In the neurodegenerative protein misfolding disorder Parkinson’s disease, the main components of cytoplasmic inclusions known as Lewy bodies are ubiquitin^17^, α-synuclein^18^,^19^ and synphilin-1 (an important α-synuclein-interacting protein)^19^,^20^. Aggregation of α-synuclein is accelerated by cationic molecules, such as glycosyl amines, polylysine and multivalent metal ions^21^, and iron is detected in Lewy bodies^22^. However, the impact and localization of NPs into cytoplasmic inclusions in neurons is not well understood.

Biogenic polyamines, such as putrescine, spermidine and spermine, are scavengers of ROS^23^. They are closely related to the biochemical activity and factors responsible for the development of neurological diseases. Cellular polyamines promote the aggregation and fibrillization of α-synuclein *in vitro* by binding to the negatively charged acidic region of its C-terminus^24^. The polyamine content is indicative of disturbances in cellular processes and can be used as a biomarker for early stage neurodegenerative diseases^25^.

In this study, the effect of MNPs@SiO_2_(RITC) was investigated in HEK293 cells, human neuroblastoma SH-SY5Y cells and primary neurons. A comprehensive approach to evaluate MNPs@SiO_2_(RITC)-induced toxicity was employed by assessing gene expression, protein aggregation, and metabolic changes.

## Results

### Altered expression of proteasome-related genes in cells treated with MNPs@SiO_2_(RITC)

We assessed the effect of exposure to 0.1 or 1.0 μg/μl MNPs@SiO_2_(RITC) for 12 h in HEK293 cells on UPS-related genes using microarray expression analysis and MultiExperiment Viewer (MeV) software. When 0.1 μg/μl MNPs@SiO_2_(RITC)-treated cells were compared to non-treated controls, the expression level of 15 UPS-related genes were found to be changed (Supplementary Fig. S1). However, when 1.0 μg/μl MNPs@SiO_2_(RITC)-treated cells were compared to non-treated controls, the expression of a total of 48 UPS-related genes were differentially expressed by >1.25-fold, including all 15 altered by 0.1 μg/μl MNPs@SiO_2_(RITC).

**Figure 1:**
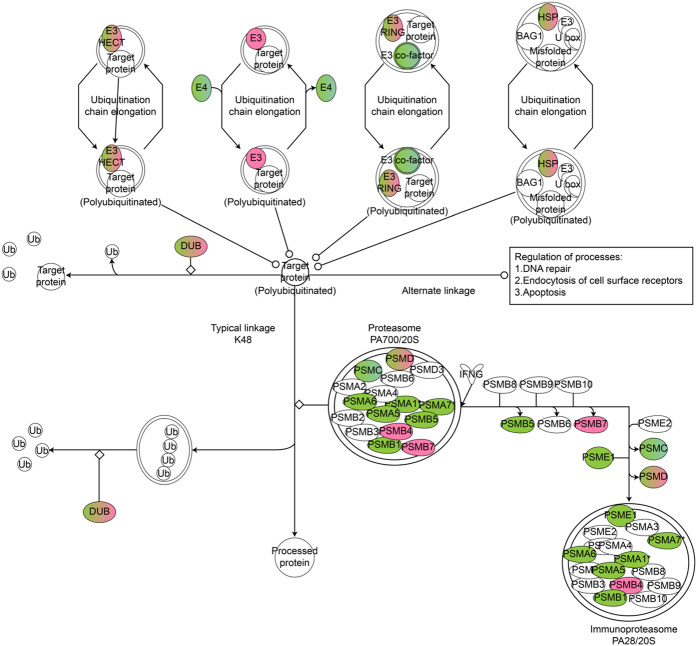
Ubiquitin proteasome pathway genes showing significantly altered expression by MNPs@SiO_2_(RITC) on microarray analysis. Ubiquitin proteasome pathway-related genes were constructed algorithmically using Ingenuity Pathway Analysis (IPA). Red and green in the genetic network indicate up- and down-regulated genes, respectively, in HEK293 cells treated with 1.0 μg/μl MNPs@SiO_2_(RITC) compared with non-treated controls for 12 h. Data set of differentially expressed genes obtained from microarray data with >1.25-fold change is shown.

**Figure 2:**
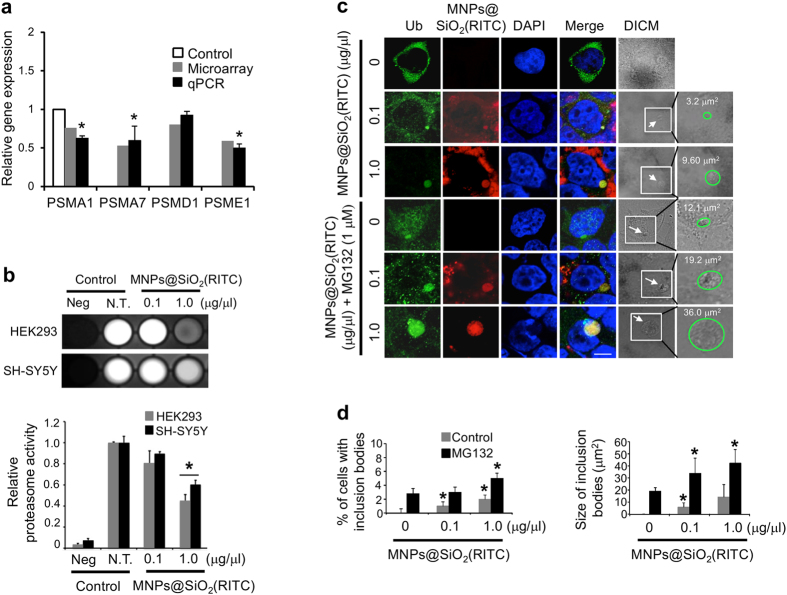
Proteasome activity and formation of inclusion bodies in HEK293 cells treated with MNPs@SiO_2_(RITC). (**a**) Quantitative analysis of ubiquitin proteasome pathway-related genes using quantitative real-time PCR. HEK293 cells were treated with 1.0 μg/μl of MNPs@SiO_2_(RITC) for 12 h. qPCR was performed using specific primers for target genes PSMA1, PSMA7, PSMD1, and PSME1. Gene expression levels of the target genes were normalized relative to the corresponding means in non-treated controls and compared with the microarray signal intensity of respective genes. GAPDH was used as internal control in qPCR. (**b**) Inhibition of proteasome activity in HEK293 and SH-SY5Y cells treated with MNPs@SiO_2_(RITC). A luminescence-based assay was performed on untreated (N.T.), 0.1 μg/μl, and 1.0 μg/μl MNPs@SiO_2_(RITC)-treated cells. The intensities were quantified with MultiGauge 3.0 software. (**c**) Characterization of ubiquitin-positive inclusion bodies in Synph-293 cells treated with MNPs@SiO_2_(RITC). Cells were treated with 0.1 μg/μl or 1.0 μg/μl of MNPs@SiO_2_(RITC) for 48 h followed by immunocytochemistry. The proteasome inhibitor MG132 (1.0 μM) was added to the cells at 36 h. Cells showed formation of cytoplasmic inclusions (arrow). Green, ubiquitin; red, MNPs@SiO_2_(RITC); blue, DAPI. Scale bar = 5 μm. (**d**) Quantification of percentage and size of ubiquitin-MNPs@SiO_2_(RITC)-positive inclusion bodies in Synph-293 cells treated with MNPs@SiO_2_(RITC). In total, 300 cells per experimental group were counted in 3 independent sets of experiments, and data represent mean values ± S.D. of triplicate measurements relative to control. *p value < 0.05 in one-way ANOVA compared to control.

Ingenuity Pathway Analysis (IPA) was used to construct a gene co-expression network from these microarray data. In cells treated with 1.0 μg/μl MNPs@SiO_2_(RITC), several UPS-related genes were significantly altered (Fig. 1, Supplementary Table S1). For example, various proteasome subunit genes, which are required for proper UPS functioning, were significantly altered. Quantitative real-time PCR (qPCR) of select proteasome subunit genes revealed significant reductions in the expression of PSMA1, PSMA7 and PSME1 (Fig. 2a). PSMD1 showed a similar tendency, although the result was not significant. Down regulation of these genes was also observed in HEK293 cells treated with silica NPs (*i.e.*, the shell of MNPs@SiO_2_(RITC)) (Supplementary Fig. S2).

### Impaired proteasome activity and formation of inclusion bodies in cells treated with MNPs@SiO_2_(RITC)

We evaluated the effect of MNPs@SiO_2_(RITC) on proteasome activity in HEK293 and SH-SY5Y cells. When 1.0 μg/μl MNPs@SiO_2_(RITC)-treated cells were compared to non-treated control cells, proteasome activity was dramatically decreased by about 40–50%, whereas 0.1 μg/μl MNPs@SiO_2_(RITC)-treated cells showed no significant difference compared to non-treated controls (Fig. 2b).

Next, Synph-293 cells were treated with 0.1 or 1.0 μg/μl MNPs@SiO_2_(RITC) for 48 h. Immunocytochemical analysis revealed staining of inclusion bodies that co-localized with MNPs@SiO_2_(RITC), with a dose-dependent increase in the frequency and size of inclusions (Fig. 2c), while less than 1% of non-treated control cells had inclusions. Specifically, among low dose MNPs@SiO_2_(RITC)-treated cells, 1% had inclusions with an average size of 5.98 μm^2^, and among high dose-treated cells 2% had inclusions with an average size of 14.24 μm^2^ (Fig. 2d). Synph-293 cells treated with MG132 also had MNPs@SiO_2_(RITC)-induced dose-dependent increases in the frequency and size of inclusion bodies: 3% of low-dose treated cells had inclusions with an average size of 33.77 μm^2^, and 5% of high dose-treated cells had inclusions with an average size of 42.16 μm^2^. Similar results were observed with lactacystin treatment (Supplementary Fig. S3). Smaller aggregate-like inclusions with diameters ranging from ~0.5–2.5 μm were also detected^26^, but could not be quantified due to their small size and low abundance.

### The impact of the silica shell of MNPs@SiO_2_(RITC) on cellular homeostasis

**Figure 3:**
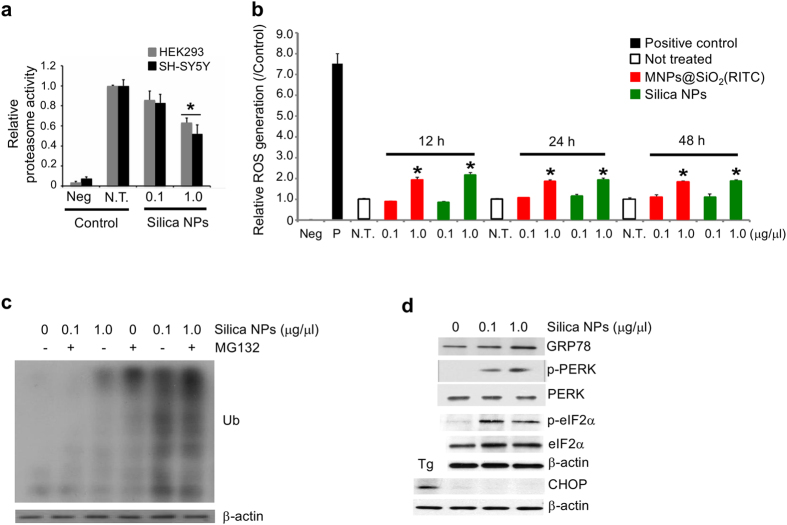
Generation of intracellular effects after treatment with MNPs@SiO_2_(RITC) and silica NPs. (**a**) Inhibition of proteasome activity in HEK293 and SH-SY5Y cells treated with silica NPs. A luminescence-based assay was performed on untreated, 0.1 μg/μl, and 1.0 μg/μl silica NPs-treated HEK293. (**b**) ROS generation in MNPs@SiO_2_(RITC)- and silica NPs-treated HEK293 cells. Cells were treated with 0.1 μg/μl or 1.0 μg/μl of MNPs@SiO_2_(RITC) or silica NPs for 12, 24, and 48 h, followed by fluorescence measurement after staining with DCFH-DA. Intensities were normalized relative to non-treated control. One mM of hydrogen peroxide (H_2_O_2_) was used as positive control and media only was used as negative control. Data represent means values ± S.D. of triplicate measurements relative to the non-treated control. (**c**) Accumulation of ubiquitinated proteins in silica NPs-treated cells. HEK293 cells were treated with 0.1 μg/μl and 1.0 μg/μl of silica NPs for 36 h, followed by treatment with MG132 or vehicle for 12 h. (**d**) ER stress-mediated apoptosis did not occur in silica NPs-treated cells. HEK293 cells were treated with 0.1 μg/μl or 1.0 μg/μl of silica NPs for 12 h, and the ER stress-related proteins GRP78, phosphorylated PERK (p-PERK), PERK, phosphorylated eIF2α (p-eIF2α), eIF2α, and CHOP were analyzed by western blotting. Ten μM Thapsigargin (Tg) was used as positive control. β-actin was used as loading control.

In earlier work, we found that the biological effects of MNPs@SiO_2_(RITC) were caused by the silica shell rather than the cobalt ferrite core when treating cells for 12 h^12^. According to another study, release of free iron in the intracellular environment from SiO_2_ coated Fe_3_O_4_ NPs induced ROS in cells^27^. Therefore, we have investigated the effects of both cobalt ferrite core and silica shell NPs in treated cells. Treating cells with 1.0 μg/μl silica NPs, but not 0.1 μg/μl, for 12 h resulted in a reduction of proteasome activity (Fig. 3a) similar to MNPs@SiO_2_(RITC)-treated cells. However, we could not exclude possible effects of the cobalt ferrite core in cells treated for a longer period of time. To address this, we compared ROS generation induced by the silica shell and the cobalt ferrite core. HEK293 cells were treated for 12, 24 or 48 h with MNPs@SiO_2_(RITC), silica NPs, or a cobalt ferrite mixture at concentrations similar to the cobalt ferrite core in the low and high doses of MNPs@SiO_2_(RITC) (Fig. 3b). As observed previously, the cobalt ferrite mixture induced high levels of ROS and cell death at both low and high concentrations^12^. Cells treated with high concentration of MNPs@SiO_2_(RITC) or silica NPs had significantly higher ROS levels compared with cells treated with a low dose, while low dose MNPs@SiO_2_(RITC) or silica NPs-treated cells did not differ from controls. Notably, ROS levels were similar between the MNPs@SiO_2_(RITC) and silica NP treatment groups. These findings suggest that the increase in ROS is due to the silica shell of MNPs@SiO_2_(RITC).

Next, we assessed the accumulation of ubiquitinated proteins in response to reduced proteasome activity. HEK293 cells were treated with 0.1 or 1.0 μg/μl silica NPs for 48 h, and ubiquitinated proteins were analyzed. Levels of ubiquitinated proteins were significantly higher in cells treated with high dose silica NPs compared with low dose or control cells (Fig. 3c). When cells were treated with silica NPs for 36 h followed by treatment with MG132 or vehicle for 12 h, a dose-dependent increase in ubiquitinated proteins was also observed.

To investigate the effect of silica NPs on ER homeostasis, we measured the expression of ER stress-related molecules (Fig. 3d). HEK293 cells treated with 0.1 or 1.0 μg/μl silica NPs for 12 h showed a dose-dependent increase in the expression of GRP78, a resident protein of the ER. The phosphorylated protein levels of PERK at Thr981 and eIF2α at Ser51 were also significantly increased by silica NPs, while their non-phosphorylated forms were not altered. However, silica NPs had no effect on the expression of CHOP proteins involved in ER stress-induced apoptosis, implying that ER stress-mediated apoptosis does not occur in cells treated with silica NPs.

### Susceptibility and formation of inclusion bodies in primary neurons treated with MNPs@SiO_2_(RITC)

**Figure 4:**
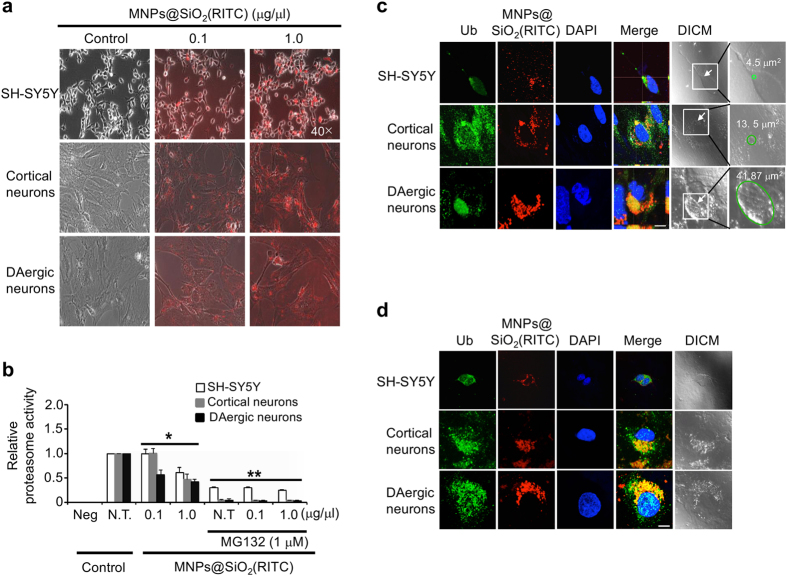
Susceptibility and formation of inclusion bodies in MNPs@SiO_2_(RITC)-treated neuronal cells. (**a**) Optical and fluorescence microscopic appearance of MNPs@SiO_2_(RITC)-treated SH-SY5Y cells, primary cortical and dopaminergic neurons. (**b**) Reduction of proteasome activity in MNPs@SiO_2_(RITC)-treated neuronal cells. To evaluate proteasome activity, SH-SY5Y cells and primary cortical and dopaminergic neurons were treated with 0.1 μg/μl and 1.0 μg/μl of MNPs@SiO_2_(RITC) for 12 h, followed by evaluation of proteasome activity by luminescence-based assay. Quantified intensities of chymotrypsin-like activity of cellular proteasomes measured using a luminometer. DAergic neurons, dopaminergic neurons. Data represent mean values ± S.D. related to the control of three independent experiments. ^*^p value < 0.05, ^**^p value* < *0.01 in one-way ANOVA compared to control were considered significantly different. (**c**) Formation of ubiquitin and MNPs@SiO_2_(RITC)-positive inclusion bodies (arrow) in SH-SY5Y cells and primary cortical and dopaminergic neuronal cells treated with 1.0 μg/μl of MNPs@SiO_2_(RITC). Z-stacks of images were acquired using NIS-Elements Advanced Research software (Nikon Instruments, Japan). (**d**) Formation of ubiquitin and MNPs@SiO_2_(RITC)-positive aggregates (arrow) in cells treated with 1.0 μg/μl of MNPs@SiO_2_(RITC). Cells were treated without or with 1.0 μg/μl of MNPs@SiO_2_(RITC) for 48 h followed by immunocytochemistry. Green, ubiquitin; red, MNPs@SiO_2_(RITC); blue, DAPI. Scale bar = 10 μm.

Rat primary cortical and dopaminergic neurons, and human SH-SY5Y cells were treated with 0.1 or 1.0 μg/μl MNPs@SiO_2_(RITC) for 12 h. Optical and fluorescence microscope images revealed a dramatic reduction in cell density in the 1.0 μg/μl MNPs@SiO_2_(RITC) treated primary cortical and dopaminergic neurons compared with untreated control cells (Fig. 4a). However, no change in cell density was observed in SH-SY5Y cells.

Similarly, in the MTS assay, primary neurons were found to be more vulnerable to MNPs@SiO_2_(RITC) than SH-SY5Y cells. In cortical neurons, high dose MNPs@SiO_2_(RITC) caused a ~50% reduction in cell viability, while the low dose had no effect. In dopaminergic neurons, significant reductions were observed with both the low dose (~40% reduced viability) and high dose (~80% reduced viability) of MNPs@SiO_2_(RITC) (Supplementary Fig. S4a).

The effect of MNPs@SiO_2_(RITC) on ROS generation was assessed next. Primary cortical and dopaminergic neurons, and SH-SY5Y cells were treated with 0.1 or 1.0 μg/μl MNPs@SiO_2_(RITC), and ROS generation was evaluated. For all three cell types, the concentration of ROS was significantly higher in the high-dose treated cells compared with the low-dose or control cells (Supplementary Fig. S4b). Additionally, in the high-dose treated cells, ROS concentration was greater in primary dopaminergic neurons than in SH-SY5Y cells and cortical neurons. Collectively, these data indicate that MNPs@SiO_2_(RITC) toxicity is mediated by ROS generation, and that primary neurons, and particularly dopaminergic neurons, are more sensitive to NPs than SH-SY5Y cells.

Primary cortical and dopaminergic neurons, and SH-SY5Y cells were treated with 0.1 or 1.0 μg/μl MNPs@SiO_2_(RITC) for 12 h. For all three cell types, proteasome activity was significantly reduced with high-concentration nanoparticle treatment relative to controls (Fig. 4b). In dopaminergic neurons, low concentration MNPs@SiO_2_(RITC) significantly reduced proteasome activity by ~40–50%. However, no significant differences were found with the low concentration in SH-SY5Y cells or primary cortical neurons. Treatment of neurons with 1.0 μM MG132 also caused a dose-dependent decrease in proteasome activity.

To determine if the NP-induced reduction in proteasome activity influences the formation of inclusion bodies in neurons, cells were treated with 0.1 or 1.0 μg/μl MNPs@SiO_2_(RITC) for 48 h. In control cells, immunocytochemical analysis revealed the presence of ubiquitin but no inclusion bodies (Supplementary Fig. S5a). On the other hand, dopaminergic neurons treated with low concentration MNPs@SiO_2_(RITC) had typical cytoplasmic inclusions that stained for ubiquitin and MNPs@SiO_2_(RITC), whereas SH-SY5Y cells or cortical neurons had smaller aggregates (Supplementary Fig. S5b).

Intracellular protein aggregates were categorized as inclusion bodies (Fig. 4c) and as aggregates (Fig. 4d) based on *size criteria*^11^. To quantify inclusion bodies, cells were counted in five to seven randomly selected areas on each cover slip by two blind investigators. A total of 130–160 cells per experimental group were counted from three independent sets of experiments. Among primary cortical neurons, 2.4% and 3.6% showed formation of inclusion bodies at low and high doses, respectively. In case of DAergic neurons, 3.6% and 5.4% had inclusion bodies in their cytosol at low and high doses, respectively (data not shown). Inclusion bodies were significantly larger in neurons treated with high dose MNPs@SiO_2_(RITC) (Fig. 4c). Cortical neurons had inclusion bodies with an average size of 3.9 μm^2^ and 12.9 μm^2^at low and high dose, respectively. Whereas, the average size of inclusion bodies in DAergic neurons was 4.2 μm^2^ and 23.2 μm^2^ at low and high dose, respectively.

The largest inclusion body, found in dopaminergic neurons, was 41.87 μm^2^. Similar to Synph-293 cells, inclusion bodies were mostly spherical and were located in the cytoplasm. Smaller aggregates (~0.5–2.5 μm) and aggresome-like inclusions (5–8 μm) were also more abundant in the high-dose treated primary dopaminergic neurons, compared with SH-SY5Y cells and cortical primary neurons (Fig. 4d). We also observed irregular inclusions with unclear boundaries in dopaminergic neurons (Supplementary Fig. S6).

### Alterations of polyamines in neuronal cells treated with MNPs@SiO_2_(RITC)

**Figure 5:**
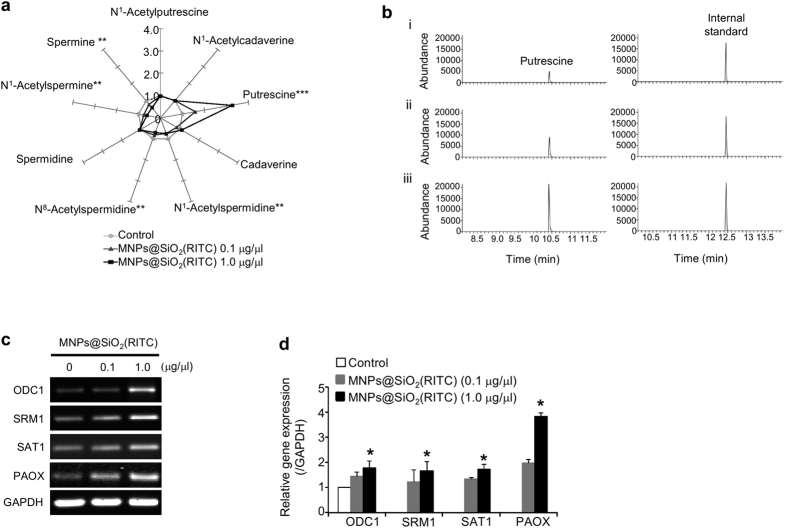
Polyamine alterations in neuronal cells treated with MNPs@SiO_2_(RITC). (**a**) Star symbol plot of composition levels of the nine polyamines in MNPs@SiO_2_(RITC)-treated SH-SY5Y cells. Star symbol plots of 0.1 μg/μl and 1.0 μg/μl of MNPs@SiO_2_(RITC)-treated cells based on the mean percentage composition levels of the nine polyamines after normalization relative to the corresponding mean values of each polyamine in the control untreated group. ^*^p value < 0.05, ^**^p value < 0.01, ^***^p value < 0.001 in one-way ANOVA compared to control were significantly different. (**b**) Selected-ion monitoring (SIM) chromatograms of putrescine in (i) untreated SH-SY5Y cells and in SH-SY5Y cells treated with (ii) 0.1 μg/μl and (iii) 1.0 μg/μl of MNPs@SiO_2_(RITC). IS, Internal standard (1.6-diaminohexane). (**c,d**) Quantitative evaluation of polyamine pathway-related genes by semiquantitative (RT-PCR) and quantitative (real-time PCR) analysis. SH-SY5Y cells were treated with 0.1 μg/μl and 1.0 μg/μl of MNPs@SiO_2_(RITC) for 12 h. RT-PCR (**c**) and real-time PCR (**d**) were performed using gene-specific primer pairs for the genes ODC1, SRM1, SAT1, and PAOX. GAPDH was used as the internal control. The histogram shows ratios of target genes, normalized relative to corresponding means in the controls. PCR products were normalized relative to internal control GAPDH. *p value < 0.05 in one-way ANOVA compared to control were significantly different. Data represent mean values ± S.D. related to control of three independent experiments.

The effect of MNPs@SiO_2_(RITC) on cellular polyamines was investigated in SH-SY5Y cells treated with 0.1 or 1.0 μg/μl MNPs@SiO_2_(RITC) for 12 h. The percentage compositions of nine polyamine metabolites were determined by GC-MS, and values were normalized to the mean level of the corresponding control. A visual star symbol plot was drawn using the resultant normalized values using rays of the plot based on Supplementary Table S2 (Fig. 5a), readily distinguishable from the nonagon shape for the non-treated control group mean. Analysis of polyamine composition revealed a significant increase in putrescine (~300%) and a significant downregulation (~30%) of *N*^*1*^–acetylspermidine, *N*^*8*^–acetylspermidine, *N*^*1*^–acetylspermine and spermine in cells treated with 1.0 μg/μl MNPs@SiO_2_(RITC). In representative selected-ion monitoring chromatograms, the peak area ratios of putrescine relative to the internal standard were about 0.29 (control; Fig. 5b,i), 0.51 (0.1 μg/μl treatment; Fig. 5b,ii), and 0.95 (1.0 μg/μl treatment; Fig. 5b,iii).

To investigate the mechanisms involved in the accumulation of putrescine, semi-quantitative reverse transcription PCR (Fig. 5c) and quantitative real-time PCR (Fig. 5d) were used to quantify gene expression of polyamine-related enzymes. Expression levels were significantly increased in cells treated with 1.0 μg/μl MNPs@SiO_2_(RITC). ODC1 and SRM1 are anabolic enzymes, while SAT1 and PAOX are catabolic enzymes. Increased levels of ODC1, SAT1 and PAOX indicate an accumulation of putrescine in SH-SY5Y cells, which agreed with the results of the GC-MS analysis. Expression of SRM1, which catalyzes the conversion of putrescine to spermidine, was lower than that of PAOX and SAT1. This suggests that the rate of conversion of putrescine to spermidine by SRM1 is slower than the rate of spermidine reconversion to putrescine by SAT1 and PAOX, resulting in the accumulation of putrescine.

## Discussion

The present study utilized a combinatorial approach of molecular biology, transcriptomics and metabolomics yielding important insights into the mechanisms of toxicity of nanoparticles to neurons.

The increase in ROS generation by MNPs@SiO_2_(RITC) in neuronal cells is consistent with our previous finding in HEK293 cells, which was linked to mitochondrial dysfunction and reduced ATP generation^12^, and is closely related to decreased proteasome activity^28^. Excessive ROS production also increases protein aggregation due to oxidative modification of proteins and reduced ATP generation^29^. Additionally, TiO_2_ NPs cause increased α-synuclein aggregation due to UPS dysfunction^30^, while silk fibroin-modified chitosan NPs cause an increase in proteasome activity *via* the PI3K/AKT1/mTOR pathway in hepatic cancer cells^31^. The discrepancies among these results can be attributed to the different NPs and cell lines used.

Oxidative conditions can cause an attenuation in proteasome activity related to misfolded protein digestion *via* protein oxidation^32^. In our previous work, we found that internalized MNPs@SiO_2_(RITC) generates significant ROS in HEK293 cells^12^, and immunocytological analysis revealed co-localization of MNPs@SiO_2_(RITC) with ROS. These findings suggest that MNPs@SiO_2_(RITC)-induced generation of ROS causes an imbalance in cellular redox homeostasis, producing an oxidized state that leads to protein aggregation and subsequent formation of inclusion bodies.

Neurons are more sensitive to the cytotoxic effects of MNPs@SiO_2_(RITC) than SH-SY5Y cells, with higher levels of ROS, lower proteasome activity and lower cell viability. This is consistent with the finding that 6-hydroxydopamine induces ROS generation and cytotoxicity at a lower dose in primary dopaminergic neurons (30 μM) than in SH-SY5Y cells (100 μM)^33^. In addition, we found that primary dopaminergic neurons are more sensitive to ROS than primary cortical neurons. This may be due to a higher expression of oxidative stress-related genes in dopaminergic neurons under conditions of oxidative stress^34^, greater receptor-mediated Ca^2+^ influx driving ROS mediated signaling^35^, and less mitochondrial mass than other neuronal types^36^. Additionally, inclusion bodies and aggregates were larger and more abundant in primary dopaminergic neurons than in primary cortical neurons or SH-SY5Y cells. We propose that dopaminergic neurons are more vulnerable to ROS-induced reductions in proteasome activity due to their high intrinsic oxidative stress^34^ and failure to increase the expression of the chaperone protein HSP70 during proteolytic stress^37^.

**Figure 6:**
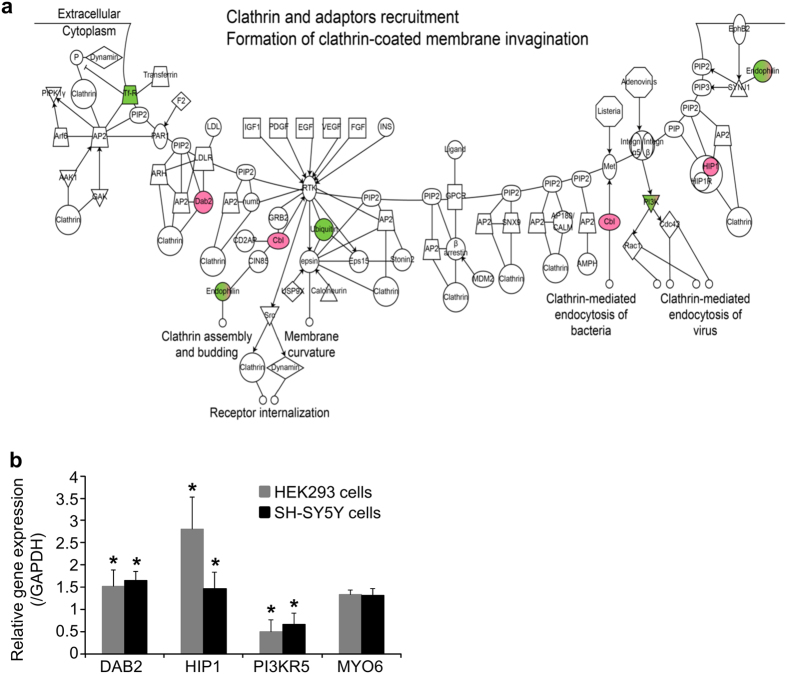
Endocytic pathway analysis in cells treated with MNPs@SiO_2_(RITC). (**a**) Clathrin-mediated endocytic pathway of genes significantly changed by MNPs@SiO_2_(RITC) on microarray analysis. HEK293 cells were treated with 1.0 μg/μl of MNPs@SiO_2_(RITC) for 12h. The pathways of endocytosis-related genes were constructed algorithmically using Ingenuity Pathway Analysis (IPA) software. Data set of differentially expressed genes was determined by microarray analysis with >1.25-fold changes. Red and green in the genetic network indicate up- and down-regulated genes in cells treated with MNPs@SiO_2_(RITC) compared with untreated cells, respectively. (**b**) Quantitative analysis of clathrin-mediated endocytosis pathway-related genes using quantitative real-time PCR. HEK293 and SH-SY5Ycells were treated with 1.0 μg/μl of MNPs@SiO_2_(RITC) for 12 h. Real-time PCR was performed using specific primer pairs for the target genes DAB2; HIP1, PI3KR5 and MYO6. GAPDH was used as internal control. Expression levels of the target genes were normalized relative to corresponding means in untreated controls and compared with the microarray signal intensity of the respective genes. *p value < 0.05 in one-way ANOVA. Data represents mean values of triplicates ± S.D. repeated independently.

MNPs@SiO_2_(RITC) were detected mainly as dot-like structures in synph-293 cells and primary neurons, but live-cell imaging techniques showed their agglomeration and sedimentation on the cell surface prior to internalization (data not shown) suggesting that these NPs enter the cell *via* endocytosis. In general, NPs smaller than 10 nm are internalized *via* the pinocytosis pathway, which functions primarily in the absorption of extracellular fluids^38^. NPs with size 10–100 nm are internalized *via* clathrin- and caveolae-mediated endocytic pathways^39^. In addition, ligand-coated nanoparticles are internalized via receptor-mediated endocytosis^40^. However, depending on the aggregation state of nanoparticles on the extracellular surface, uptake pathway may differ. For example, 50 nm silica-coated iron nanoparticle is internalized into cells *via* caveoli mediated endocytic pathway^41^ while 300 nm-sized silica nanoparticle is internalized *via* clathrin mediated endocytic pathway^42^. These discrepancies are due to non-specific binding of nanoparticles. Even though MNPs@SiO_2_(RITC) is not coated with specific ligand, we could not exclude the possibility of non-specific binding to a receptor. We believe that clathrin-mediated endocytic pathways is a major endocytosis pathway in MNPs@SiO_2_(RITC)-treated neuronal cells because caveolae-mediated endocytic pathway is absent in neurons as these cells do not express caveolin^43^. Therefore, we analyzed the clathrin-mediated endocytic pathway using microarray data in HEK293 cells. Genes related to this pathway were altered following exposure to 1.0 μg/μl MNPs@SiO_2_(RITC) (Fig. 6a, Supplementary Table S3). Real-time PCR analysis of clathrin-mediated genes in HEK293 cells and SH-SY5Y cells showed similar trends at the high concentration (Fig. 6b, Supplementary Table S4). For example, gene expression of the endocytic adapter proteins disabled homolog 2, mitogen-responsive phosphoprotein (DAB2) and huntingtin-interacting protein-1 (HIP1) was increased significantly, while expression of Phosphoinositide-3-kinase, regulatory subunit 5 (PI3KR5), which is involved in membrane re-construction during endocytosis, was decreased significantly. Gene expression of Homo sapiens myosin VI (MYO6) was not changed in microarray and real-time PCR analysis. These findings suggest that MNPs@SiO_2_(RITC) alters the expression of genes related to the clathrin-mediated endocytic pathway in neurons. Expression of various endocytic transport genes was also altered significantly (data not shown).

Cellular polyamines increase the aggregation and fibrillization of α-synuclein *in vitro* and induce formation of aggresomes due to their polycationic nature^24^. They are highly charged cationic polymers that bind to α-synuclein *via* strong electrostatic and hydrophobic bonds^24^. The isoelectric points (pI) of the inclusion body constituent proteins α-synuclein, synphilin-1 and ubiquitin were calculated *via* ExPASy (http://www.expasy.org/) as 4.76, 5.96 and 6.79, respectively. These pI values are lower than the physiologic cytoplasmic pH (~7.0–7.4)^44^, and we hypothesize that the positively charged polyamines interact with negatively charged proteins in the cytosol to become incorporated in inclusion bodies. As spermine and spermidine are tri- and tetra-cationic, respectively, they bind more frequently to proteins that form inclusion bodies such as α-synuclein^24^. Putrescine, however, is bi-cationic and binds to these proteins at a lower affinity. This may explain the elevated putrescine in the cerebrospinal fluid of patients with Parkinson’s disease observed in our previous report^25^.

MNPs@SiO_2_(RITC) toxicity has not been detected using conventional *in vitro* or *in vivo* assays^3^,^5^. Our present findings point to the limitations of evaluating nanotoxicity using conventional techniques. This is because the effects of nanoparticles are usually very small, but the accumulation of such small effects over a certain period of time can lead to cell death.

**Figure 7:**
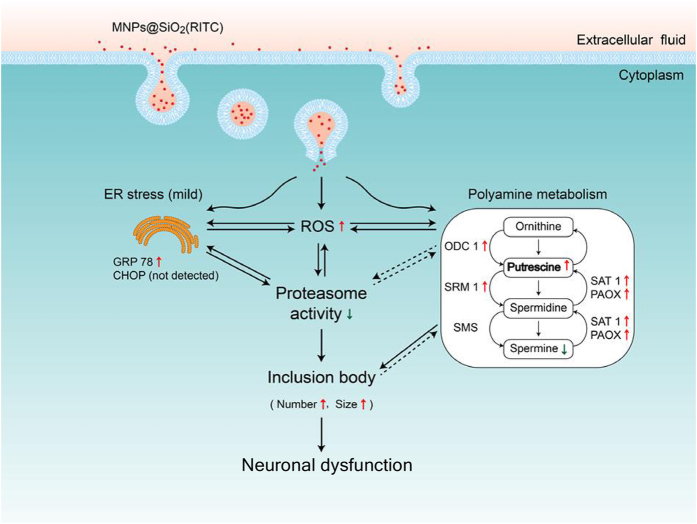
Schematic representation of cellular events that occur after treatment with high concentration MNPs@SiO_2_(RITC). Internalized MNPs@SiO_2_(RITC) induces ROS generation and disturbs cellular polyamine metabolism, but does not induce ER stress-mediated apoptosis in immortalized cell lines. Especially, elevated ROS reduces proteasome activity and induces the formation of cytoplasmic inclusions in neuronal cells. Solid arrows denote known functional relationships based on the present and prior studies. Dotted arrows indicate mechanisms that are currently unknown.

We propose three aspects of cellular events that occur following treatment with a high dose of MNPs@SiO_2_(RITC) in neurons (Fig. 7). (i) MNPs@SiO_2_(RITC) induces ROS generation, which leads to a reduction in UPS activity. This results in a dose-dependent formation of inclusion bodies that varies in frequency and size. (*ii*) Internalized MNPs@SiO_2_(RITC) leads to cell death in primary neurons, but does not induce CHOP in HEK293 cells, suggesting that ER stress is not a primary trigger of apoptosis in this cell line. Elevation of ER stress may be related to increased ROS generation and reduced proteasome activity in MNPs@SiO_2_(RITC) treated cells, as these phenomena are believed to function in a reciprocal manner^45^. (*iii*) MNPs@SiO_2_(RITC) significantly alters cellular polyamines and genes related to polyamine metabolism leading to increased inclusion body formation and neuronal dysfunction. Although the relevance of MNPs@SiO2(RITC) and their cytotoxic effects are well recognized by comprehensive studies in healthy cells, there is a pressing need for testing all these parameters in stressed and diseased cells that might be more susceptible and resemble the pathological conditions for which nanoparticles might be employed for diagnostic or therapeutic applications. In addition, to clarify whether the concentrations of MNPs@SiO_2_(RITC) in culture cells relate to those achieved in the brain *in vivo* for medical diagnostic and therapeutic applications, further studies are needed to determine nanoparticle distribution using magnetic resonance imaging, inductively coupled plasma mass spectrometry, and X-ray absorption near edge structure spectroscopy.

In conclusion, the present findings suggest that exposure to high doses of NPs can be deleterious to neurons. These results highlight the critical importance of using low doses of NPs for safety considerations. It is helpful for both the therapeutic or diagnostic applications of nanoparticles to use safe dosages based on our sensitive and comprehensive evaluation of their toxicity in neurons. Further *in vivo* studies evaluating the molecular mechanisms of inclusion formation and the alteration of polyamine metabolites in NP-treated neurons will help improve our understanding of neuronanotoxicology and aid in the development of safe NPs as neural therapeutic and diagnostic agents.

## Materials and Methods

### Cell culture

Human embryonic kidney 293 (HEK293) cells and human neuroblastoma SH-SY5Y cells were obtained from American Type Culture Collection (ATCC). HEK293 cells over-expressing FLAG-tagged synphilin-1 (synph-293 cells) were used to evaluate the formation of cytoplasmic inclusions, because the probability of forming such inclusions is high^20^,^46^. HEK293 cells are similar to established neuronal lineage cell lines (NTERA-2 cells) and show strong staining for neurofilament (NF)-specific antibodies such as NF-M, NF-L, and NF-H^47^. Cells were cultured in Dulbecco’s high-glucose modified Eagle’s medium (DMEM, Gibco, USA) supplemented with 10% fetal bovine serum (Gibco, USA), 100 units/ml penicillin, and 100 μg/ml streptomycin (Gibco, USA) and incubated in a 5% humidified CO_2_ chamber at 37 °C.

### Primary neuronal cell culture

Primary cortical and dopaminergic (DAergic) neurons (Day *in vitro* 20–23) were prepared from 1-day-old Sprague–Dawley rats as described elsewhere^48^. To obtain pure neuronal cultures, glial cells were removed by mechanical shaking, followed by washing. Purity of the cultures was verified by 99.9% of cortical and dopaminergic neurons staining with specific antibodies to neuronal nuclei (NeuN) and tyrosine hydroxylase (TH), respectively (Supplementary Fig. S7).

### MNPs@SiO_2_(RITC) and silica NPs

MNPs@SiO_2_(RITC) particles contain a cobalt ferrite core (CoFe_2_O_3_) sheathed by a silica shell that is chemically bonded to rhodamine isothiocyanate dye (RITC)^49^. Silica NPs are identical to MNPs@SiO_2_(RITC) except that they lack a cobalt ferrite core and show similar tendencies with respect to their biological effects^12^,^50^. MNPs@SiO_2_(RITC) and silica NPs are 50 nm in diameter, and the size distribution and *zeta*-potential of both NPs have been previously reported^49^,^50^. A previous study indicated that approximately 10^5^ particles of MNPs@SiO_2_(RITC) per cell were taken up in MCF-7 breast cancer cells as determined by inductively coupled plasma atomic emission spectrometry (ICP-AES)^49^. The dosage used in this study was determined by treating HEK293 cells with MNPs@SiO_2_(RITC) at concentrations ranging from 0.01 to 2.0 μg/μl for 12 h and calculating their uptake efficiencies using a fluorescent assessment method^12^. The optimal concentration of MNPs@SiO_2_(RITC) was 0.1 μg/μl for *in vitro* use and as MRI contrast without toxicological effects in human cord blood-derived mesenchymal stem cells^3^. Disturbances of gene expression and metabolic profiles at this concentration were similar to those in control HEK293 cells^12^. The uptake efficiency of MNPs@SiO_2_(RITC) plateaued at 1.0 μg/μl. Therefore, a low dose of 0.1 μg/μl and high dose of 1.0 μg/μl were used in the present study.

### Immunocytochemistry

Cells were treated with MNPs@SiO_2_(RITC) for 36 h, followed by treatment with the proteasome inhibitor MG132 or vehicle for 12 h. The cells were then fixed in 4% paraformaldehyde solution for 30 min, dehydrated, and permeabilized in 0.3% Triton-X100 for 15 min. This was followed by overnight incubation at 4 °C with primary antibodies (ubiquitin, TH and NeuN; 1:200; Santa Cruz Technologies, USA). Cells were then incubated with fluorescein-conjugated secondary antibodies for 1 h at room temperature, followed by washing with phosphate-buffered saline (PBS). Cover slips were mounted onto slides using mounting medium with 4′,6-diamidino-2-phenylindole (DAPI). Fluorescent images were acquired by confocal laser scanning microscopy (LSM) (Nikon, Japan). The excitation wavelengths for fluorescein, DAPI and MNPs@SiO_2_(RITC) were 494, 405 and 530 nm, respectively. NIS-Elements Advanced Research software was used to acquire all digital images (Nikon Instruments, Japan). To quantify the formation of ubiquitin-MNPs@SiO_2_(RITC) inclusion bodies, LSM images were combined with differential interference contrast microscopy (DICM) images. We investigated whether MNPs@SiO_2_(RITC)-induced impairment of proteasome activity influences the formation of inclusion bodies in HEK293 cells stably expressing FLAG-tagged synphilin-1 (Synph-293 cells). In an analysis of inclusion bodies formed in MNPs@SiO_2_(RITC)-treated cells, larger inclusions were observed with ubiquitin antibody than with FLAG antibody used to detect synphilin-1 (Supplementary Fig. S8). For this reason, ubiquitin antibody was used for subsequent immunocytochemical analyses of inclusions. Cells were counted in five to seven randomly chosen areas on each cover slip by two blind investigators. A total of 300 cells per experimental group were counted from three independent sets of experiments, as described previously^20^,^46^.

### Gas chromatography-mass spectrometry

Gas chromatography-mass spectrometry (GC-MS) was used to determine the levels of various polyamines in MNPs@SiO_2_(RITC)-treated cells. The polyamines putrescine, spermidine, spermine, *N*^*1*^-acetylputrescine, *N*^*1*^-acetylcadaverine, *N*^*1*^-acetylspermidine, *N*^*8*^-acetylspermidine, *N*^*1*^-acetylspermine, and cadaverine as well as the internal standard (IS) 1,6-diaminohexane were from Sigma-Aldrich (USA). GC-MS analyses were performed using an Agilent 6890N gas chromatograph interfaced with an Agilent 5975B mass-selective detector and an ultra cross-linked capillary column (Agilent Technologies, USA)^12^. Analyses were performed in both scan and selected-ion monitoring (SIM) modes. The mass range scanned was 50 to 600 u at a rate of 0.99 scans/s for analyses in scan mode. For SIM mode, three characteristic ions for each polyamine were used for peak identification and quantification as described elsewhere^25^.

### Statistical analysis

The results were analyzed by one-way analysis of variance (ANOVA) using IBM-SPSS software (IBM Corp., USA). In all analyses, *p* value < 0.05 was taken to indicate statistical significance.

## Additional Information

**How to cite this article**: Phukan, G. *et al*. Silica-coated magnetic nanoparticles impair proteasome activity and increase the formation of cytoplasmic inclusion bodies *in vitro. Sci. Rep.* **6**, 29095; doi: 10.1038/srep29095 (2016).

## Supplementary Material

Supplementary Information
