# Supplementary material for: Silica-coated magnetic nanoparticles impair proteasome activity and increase the formation of cytoplasmic inclusion bodies in vitro
Source: Sci Rep. 2016 Jul 5;6:29095. doi: 10.1038/srep29095 (PMC4932509; doi:10.1038/srep29095)
Supplement: Supplementary Information [file srep29095-s1.doc]

**Silica-coated magnetic nanoparticles impair proteasome activity and increase the formation of cytoplasmic inclusion bodies *in vitro***

Geetika Phukan^1#^, Tae Hwan Shin^1,2#^, Jeom Soon Shim^1^, Man Jeong Paik^3^, Jin-Kyu Lee^4^, Sangdun Choi^2^, Yong Man Kim^5^, Seong Ho Kang^6^, Hyung Sik Kim^7^, Yup Kang^1^, Soo Hwan Lee^1^, M. Maral Mouradian^8^, Gwang Lee^1*^

*^1^Department of Physiology and Department of Biomedical Sciences, Ajou University School of Medicine, Suwon, Republic of Korea*

*^2^ Department of Molecular Science and Technology, Ajou University, Suwon, Republic of Korea*

*^3^College of Pharmacy, Sunchon National University, Suncheon, Republic of Korea*

*^4^Department of Chemistry, Seoul National University, Seoul, Republic of Korea.*

^5^*Pharmicell Co., Ltd. Sungnam, Republic of Korea*

*^6^Department of Applied Chemistry and Institute of Natural Sciences, Kyung Hee University, Yongin-si, Republic of Korea*

*^7^School of Pharmacy, Sungkyunkwan University, Suwon, Republic of Korea*

*^8^Center for Neurodegenerative and Neuroimmunologic Diseases, Department of Neurology, Rutgers – Robert Wood Johnson Medical School, Piscataway, NJ, USA*

^#^: These authors contributed equally to this work.

Running title: Silica-coated magnetic nanoparticles increase cytoplasmic inclusions

^*^Corresponding author: Gwang Lee

Associate Professor, Department of Physiology

Ajou University School of Medicine, 164, World cup-ro,

Yeongtong-gu, Suwon, 443-721, Republic of Korea.

Tel: +82-31-219-4554

C.P.:+82-10-3738-9349

Fax: +82-31-219-5049

E-mail : [glee@ajou.ac.kr](mailto:glee@ajou.ac.kr), gwanglee@hanmail.net

**Supplementary information**

**Methods**

**Microarray data analysis**

Differences in gene expression of cells were examined using the Affymetrix system (ISTECH, South Korea) in conjunction with the Human U133 Plus 2.0 50K microarray, which contains 54,675 probes. Differences in the data distribution were analyzed with GenPlex 3.0 software based on our previous report[^1^](#_ENREF_1). Biological pathways and functions were identified using the web-based bioinformatics software Ingenuity Pathway Analysis (IPA version 8.5; Ingenuity Systems, USA). A 1.25-fold change in expression was used as a cut-off to generate data sets of significantly changed genes.

**RNA purification**

Total RNA was extracted from MNPs@SiO_2_(RITC)-treated or non-treated cells using RNAZol B (Tel-Test, Inc., USA) and then purified using an RNeasy Mini kit (Qiagen, Germany) according to the manufacturers’ instructions. Briefly, 2 × 10^6^ cells were harvested with 500 μl of RNAZol B solution, and 70 μl of chloroform was added. This was followed by incubation for 5 min on ice. Cells were then treated with 600 μl of isopropyl alcohol to precipitate RNA. Pellets were then washed in 70% ethanol followed by air-drying, and RNA was then eluted with RNase-free water. The purity of RNA was determined by spectrophotometry (Eppendorf, USA). RNA with optical density values of 1.8 to 2.0 at 260/230 and 260/280 was used for PCR experiments.

**Quantitative real-time PCR (qPCR) and reverse transcription PCR (RT-PCR)**

To quantify gene expression, total RNA samples were reverse transcribed using a RealMOD SYBRGreen real-time PCR kit (iNtRON Biotechnology, South Korea) with gene-specific primers. The primer sequences for specific genes are listed in Supplementary Table S5 (proteasome subunit real-time primers), Supplementary Table S6 (proteasome subunit RT-PCR primers), and Supplementary Table S7 (polyamine enzyme real-time primers and RT-PCR primers). Reaction conditions were 5°C for 2 min, 95°C for 30 s, followed by 40 cycles at 95°C for 5 s and 53°C for 30 s. The threshold/quantification cycle (C_t_/C_q_) value was determined as the point at which fluorescence increased significantly above the background level. A melting curve was generated to analyze the PCR products. PCR was run in independent triplicates. The relative quantification of gene expression was performed using the 2^-ddCt^ method.

For semi-quantitative RT-PCR, total RNA samples were reverse transcribed and amplified using gene-specific primer pairs for genes related to the proteasome and polyamine metabolic pathway. To remove template errors, PCR products were normalized relative to glyceraldehyde-3-phosphate dehydrogenase (GAPDH) PCR products amplified from the same samples. Amplified PCR products were separated on 3% agarose gels and stained with ethidium bromide.

**Proteasome activity assay**

Proteasome activity was analyzed using Proteasome-Glo™ Chymotrypsin-Like Cell Based Assay (Promega, USA) according to the manufacturer’s protocol. Briefly, HEK293, SH-SY5Y, and primary neuronal cells were seeded onto 96-well plates at a density of 2.5 × 10^4^ cells per well and incubated at 37°C in a 5% CO_2_ humidified chamber. Cells were treated with MNPs@SiO_2_(RITC) and silica NPs for 12 h. Cells were then washed three times with PBS, replenished with 50 μl of serum-free media, and kept at room temperature. Proteasome-Glo™ cell-based buffer was mixed with the luciferin detection agent and appropriate Proteasome-Glo™ substrate and incubated at room temperature for 30 min. Aliquots of 50 μl of this mixture were then added to medium in each well and incubated at room temperature for 10 to 15 min. Next, 90 μl of the medium containing proteasome assay solution was transferred to corresponding wells on a white plate. Proteasome activity of each sample was measured by measuring the chymotrypsin-like activity of cellular proteasomes using the luminogenic substrate Suc-Leu-Leu-Val-Tyr-aminoluciferin with a luminometer (LMaxII^384^; Molecular Devices, USA) according to the manufacturer’s instructions. Luminescence images were acquired using the LAS-1000 Image Analysis System (Life Sciences, USA).

**MTS assay**

For the cell viability assay, an MTS assay was performed using a CellTiter 96-cell proliferation assay kit (Promega, USA) according to the manufacturer’s instructions. Briefly, aliquots of 2 × 10^4^ cells were cultured on 96-well assay plates. At confluence, cells were washed with PBS and treated with MNPs@SiO_2_(RITC) for 12 h. The cells were then washed with PBS to remove excess MNPs@SiO_2_(RITC), and 20 μl of MTS solution was added to each well of the 96-well assay plate containing treated cells in 100 μl of culture medium. The assay plate was then incubated for 1 h under 5% CO_2_ at 37°C. The amount of soluble formazan produced by cellular reduction was directly measured with a plate reader at a wavelength of 490 nm. Values were normalized relative to the protein OD value for each corresponding group.

**ROS measurement in neuronal cells**

Intracellular ROS levels in MNPs@SiO_2_(RITC), silica shell and metal core treated cells were evaluated using 2',7'-dichlorodihydrofluorescein diacetate (DCFH-DA) (Cell Biolabs, USA). To determine whether ROS generation was induced by silica shell or the metal core of MNPs@SiO_2_(RITC), HEK293 cells were treated with MNPs@SiO_2_(RITC), silica NPs, and each metal at similar concentrations of FeCl_3_·6H_2_O, CoCl_2_·6H_2_O, and Fe (NO_3_)_3_·9H_2_O in the cobalt ferrite core (CoFe_2_O_4_) based on the diameter of the cobalt ferrite core (9 nm), the density of MNPs@SiO_2_(RITC) (2.2 μg/mm^3^), and the density of the cobalt ferrite core (5.3 μg/mm^3^)[^1^](#_ENREF_1),[^2^](#_ENREF_2). To determine MNPs@SiO_2_(RITC)-induced intracellular ROS generation, SH-SY5Y, primary cortical neurons, and primary dopaminergic neurons were treated with MNPs@SiO_2_(RITC) at doses of 0.1 and 1.0 μg/μl for 12 h. Measurement of ROS generation was done in accordance with the manufacturer’s instructions. Briefly, cells were seeded onto 96-well clear cell culture plates at a density of 5 × 10^4^ cells. Cells were treated with MNPs@SiO_2_(RITC) at doses of 0.1 and 1.0 μg/μl for 12 h, washed with serum-free medium, and 1× DCFH-DA/media solution was added and incubated at 37°C for 45 min. Cells were then washed again with serum-free medium. This was followed by addition of 100 μl of medium and 100 μl of 2× cell lysis buffer to cells with thorough mixing. After incubating the cells for 10 min, 150 μl of the mixture was transferred to the corresponding well in a 96-well black plate. Fluorescence was detected using a Fluorescence Microplate Reader (Spectra MAX GEMINI EM; Molecular Devices, USA) at excitation and emission wavelengths of 480 and 530 nm, respectively. Values were normalized relative to the protein concentration of the respective samples determined by Bradford’s assay.

**Western blotting**

HEK293 cells were seeded at a density of 5 × 10^5^ cells/well in 6-well plates and cultured for 24–36 h. Cells were treated with 0, 0.1 or 1.0 µg/µl silica NPs in serum-free media, washed with PBS, and harvested using 0.25% trypsin/0.1% EDTA (Sigma, USA). Cells were then lysed in RIPA buffer along with phosphatase inhibitor (1 ml/40 mg pellet) and mixed well by pipetting. Lysates were vortexed mildly and incubated at 4^o^C for 1 h. This was followed by centrifugation at 14,000 g for 15 min at 4^o^C. The supernatant was collected, and 10–40 µg of protein was loaded onto SDS–PAGE and transferred onto nitrocellulose membranes. Membranes were blocked with 5% non-fat milk for 1 h at room temperature and incubated with primary antibody overnight at 4^o^C. Antibodies used were ubiquitin (1:500, Santa Cruz Technologies, USA), 78 kDa glucose-regulated protein (GRP78) (1:1,000, Abcam, USA), phosphorylated protein kinase RNA-like endoplasmic reticulum kinase (p-PERK) (1:500, Santa Cruz Technologies, USA), protein kinase RNA-like endoplasmic reticulum kinase (PERK) (1:500, Santa Cruz Technologies, USA), phosphorylated eukaryotic initiation factor 2 α (p-eIF2α) (1:2,000, Cell signaling, USA), eukaryotic initiation factor 2 α (eIF2α) (1:2,000, Cell signaling, USA) and C/EBP homologous protein (CHOP) (1:2,000, Cell Signaling, USA) and beta-actin (1:5,000, Cell Signaling, USA). Secondary antibodies were used at a dilution of 1:1,000-1:10,000 (Abclone, South Korea). Blots were developed using iNtron West ZolPlus enhanced chemiluminescence (ECL) solution (Intron Biotechnologies, South Korea), and luminescence was captured on medical blue X-ray film (AGFA, Belgium) in dark room.

**Star symbol plotting**

The amounts of polyamines in MNPs@SiO_2_(RITC)-treated cells were determined using calibration curves that were converted to percentage composition. This was followed by normalization of the polyamine level in each group to the mean value of the corresponding control group, and these normalized values were plotted as radiating lines emerging from a central common point. The far ends of the lines were joined to produce star-like patterns using MS Excel (Microsoft, USA)[^1^](#_ENREF_1).

**Reference**

1. Shim, W. et al. Analysis of changes in gene expression and metabolic profiles induced by silica-coated magnetic nanoparticles. *ACS Nano* **6**, 7665-7680 (2012).

2. Yoon, T.J. et al. Multifunctional nanoparticles possessing a "magnetic motor effect" for drug or gene delivery. *Angew Chem Int Ed Engl* **44**, 1068-1071 (2005).

**Tables**

**Table S1**. Microarray data-based profiles of proteasome subunit-related genes in HEK293 cells treated with MNPs@SiO_2_(RITC)

| **Gene name** | **Symbol** | **Probe ID** | **Chromosomal**  **Location** | **NCBI Ref. seq** | **Signal** | | |
| --- | --- | --- | --- | --- | --- | --- | --- |
|  |  |  |  |  | **control** | **0.1 μg/μl** | **1.0 μg/μl** |
| Proteasome subunit, alpha type 1 | PSMA1 | 201676_x_at | 11p15.1 | NM_002786.1 | 30388.8 | 26698.9 | 23074.9 |
| Proteasome subunit, alpha type 5 | PSMA5 | 201274_at | 1p13 | NM_002790.3 | 6395.6 | 6149.3 | 4752.7 |
| Proteasome subunit, alpha type7 | PSMA7 | 201114_x_at | 20q13.33 | NM_002792.3 | 19744.9 | 13668.9 | 10409 |
| Proteasome 26S subunit, non-ATPase 1 | PSMD1 | 201199_s_at | 2q37.1 | NM_002807.3 | 7316 | 6999 | 5873.5 |
| Proteasome 26S subunit, non-ATPase 7 | PSMD7 | 201705_at | 16q22.3 | NM_002811.4 | 3259.4 | 2685.9 | 2274.3 |
| Proteasome 26S subunit, non-ATPase 10 | PSMD10 | 219485_s_at | Xq22.3 | NM_170750.2 | 4464.1 | 4255.6 | 2245.9 |
| Proteasome activator subunit 1 (PA28 alpha) | PSME1 | 200814_at | 14q11.2 | NM_006263.3 | 8154.4 | 5583.1 | 4796 |

Ref. seq.: Reference sequence

**Table S2.** Composition percentage of polyamines found in control, 0.1 μg/μl and 1.0 μg/μl MNPs@SiO_2_(RITC)-treated SH-SY5Y cells

|  | **Polyamine** | **Composition ^a^** | | | |  | **Normalized value ^e^** | |
| --- | --- | --- | --- | --- | --- | --- | --- | --- |
|  |  | **Control (n=3)** | **MNPs@SiO_2_(RITC) treated group (n=3)** | | |  | **Low conc.** | **High conc.** |
|  |  |  | **Low conc. (0.1 μg/μl)** | **High conc. (1.0 μg/μl)** | ***P* value ^d^** |  |  |  |
|  | *N*^1^-Acetylputrescine | ND | ND | ND | ND |  | 1.0 | 1.0 |
|  | *N*^1^-Acetylcadaverine | ND | ND | ND | ND |  | 1.0 | 1.0 |
|  | Putrescine | 1.06 ± 0.03 | 1.67 ± 0.06 (<0.0001)^b^ | 3.45 ± 0.13 (<0.0001)^c^ | **<**0.001 |  | 1.6 | 3.3 |
|  | Cadaverine | 0.22 ± 0.02 | 0.18 ± 0.02 (0.085) | 0.24 ± 0.01 (0.287) | 0.085 |  | 0.8 | 1.1 |
|  | *N*^1^-Acetylspermidine | 17.87 ± 1.41 | 13.64 ± 1.15 (0.016) | 13.40 ± 1.25 (0.012) | 0.97 |  | 0.8 | 0.7 |
|  | *N*^8^-Acetylspermidine | 19.79 ± 1.66 | 16.18 ± 1.33 (0.045) | 13.71 ± 1.17 (0.004) | 0.158 |  | 0.8 | 0.7 |
|  | Spermidine | 23.07 ± 1.7 | 25.43 ± 0.4 (0.073) | 25.33 ± 0.45 (0.084) | 0.992 |  | 1.0 | 1.1 |
|  | *N*^1^-Acetylspermine | 16.47 ± 1.27 | 12.50 ± 0.90 (0.009) | 10.44 ± 0.98 (0.001) | 0.119 |  | 0.8 | 0.6 |
|  | Spermine | 8.67 ± 0.72 | 6.96 ± 0.45 (0.024) | 5.22 ± 0.49 (0.001) | 0.022 |  | 0.8 | 0.6 |

^a^ Values as percentage of composition of each polyamine amount relative to the total polyamine amount (μg/10^6^ cells).

^b^ One way ANOVA of the mean values of the controls and the group treated with MNPs@SiO_2_(RITC) of 0.1 μg/μl.

^c^ One way ANOVA of the mean values of control group and treated group with MNPs@SiO_2_(RITC) of 1.0 μg/μl.

^d^ One way ANOVA of the mean values of between treated 0.1 μg/μl group and 1.0 μg/μl group with MNPs@SiO_2_(RITC).

^e^ Normalized values of polyamines in treated groups with MNPs@SiO_2_(RITC) to corresponding mean values of in control group.

Low concentration: 0.1 μg/μl MNPs@SiO_2_(RITC)

High concentration: 1.0 μg/μl MNPs@SiO_2_(RITC)

ND: Not detected

**Table S3**. Microarray data-based profiles of clathrin-mediated endocytic pathway-related genes in HEK293 cells treated with MNPs@SiO_2_(RITC)

| **Gene name** | **Symbol** | **Probe ID** | **Chromosomal location** | **NCBI Ref. seq.** | **Signal** | | |  |
| --- | --- | --- | --- | --- | --- | --- | --- | --- |
|  |  |  |  |  | **Control** | **0.1 μg/μl** | **1.0 μg/μl** | |
| Transferrin receptor | TFRC | 208691_at | chr3q29 | NM_003234.2 | 4469.8 | 4110.2 | 772.8 | |
| Disabled homolog 2,mitogen-responsive phosphoprotein | DAB2 | 201280_s_at | chr5p13.1 | NM_001343.3 | 371.2 | 565.0 | 1024 | |
| Cas-Br-M (murine) ecotropic retroviral transforming sequence | CBL | 229010_at | chr11q23.3 | NM_005188.3 | 26.4 | 198.9 | 274.9 | |
| SH3-domain GRB2-like 3 (Endophilin) | SH3GL3 | 205637_s_at | chr15q24 | NM_003027.4 | 1063.1 | 1319.7 | 512.4 | |
| Phosphoinositide-3-kinase, regulatory subunit 5 | PIK3R5 | 220566_at | chr17p13.1 | NM_014308.3 | 310.2 | 101.5 | 169.4 | |
| Huntingtin interacting protein 1 | HIP1 | 205426_s_at | chr7q11.23 | NM_005338.6 | 57.2 | 245.2 | 585.3 | |
| Heat shock 70kDa protein 8 | HSPA8 | 208687_x_at | chr11q24.1 | NM_006597.5 | 42436.0 | 44917.8 | 15233.9 | |
| Homo sapiens myosin VI | MYO6 | 203215_s_at | chr6q14.1 | NM_004999.3 | 180.9 | 504.6 | 1009.2 | |
| Cortactin | CTTN | 214782_at | chr11q13 | NM_138565.2 | 164.3 | 183.0 | 670.0 | |

Ref. seq.: Reference sequence

**Table S4.** Quantitative real-time PCR primer sequences of clathrin-mediated endocytic pathway-related genes

| **Gene Name** | **Symbol** | **NCBI Ref. seq** | **Direction** | **Primer sequence (5’-3’)** |
| --- | --- | --- | --- | --- |
| Phosphoinositide-3-kinase, regulatory subunit 5 | PIK3R5 | NM_014308.3 | Forward | ACA CGT GCC ATC AAG GC |
|  |  |  | Reverse | CAC GGA GGT ACA GAC C |
| Disabled homolog 2, mitogen-responsive phosphoprotein | DAB2 | NM_001343.3 | Forward | CAC TGC CTT AGA CCC AC |
|  |  |  | Reverse | CCT GAG GAA TGC CAA CC |
| Huntingtin interacting protein 1 | HIP1 | NM_005338.6 | Forward | GCC ACT GTC ATG GTG GA |
|  |  |  | Reverse | GGT TGG GGC TGT CCT TA |
| Homo sapiens myosin VI | MYO6 | NM_004999.3 | Forward | ATT CAT CCG CCC TGC C |
|  |  |  | Reverse | TGA GGA GAC TGG CCT GA |
| Homo sapiens glyceraldehyde 3-phosphate dehydrogenase | GAPDH | NM_002046 | Forward | GAA GAC TGT GGA TGG CCC |
|  |  |  | Reverse | CCA TGC CAG TGA GCT TCC |

Ref. seq.: Reference sequence

**Table S5.** Quantitative real-time PCR primer sequences for genes encoding proteasomal subunits

| **Gene Name** | **Symbol** | **NCBI Ref. seq.** | **Direction** | **Primer sequence (5’-3’)** |
| --- | --- | --- | --- | --- |
| Proteasome subunit, alpha type 1 | PSMA1 | NM_002786.1 | Forward | GAG GGT GTG TGC CTA GCT |
|  |  |  | Reverse | CTG TGT CTC CAC TCT GGC |
| Proteasome subunit, alpha type 7 | PSMA7 | NM_002792.3 | Forward | CAG GAG GCC GTC AAG A |
|  |  |  | Reverse | CCT GCA AAG GCC ATG CA |
| Proteasome 26S subunit, non-ATPase 1 | PSMD1 | NM_002807.3 | Forward | GCC AGA GCC ACT AAC TGG |
|  |  |  | Reverse | GGC TGA TCC TGG AGA AG |
| Proteasome activator subunit 1 (PA28 alpha) | PSME1 | NM_006263.3 | Forward | AGG CCC AAG CCA AGG T |
|  |  |  | Reverse | GGG CCT TCA GAT TGC TC |
| Homo sapiens glyceraldehyde 3-phosphate dehydrogenase | GAPDH | NM_002046 | Forward | GAA GAC TGT GGA TGG CCC |
|  |  |  | Reverse | CCA TGC CAG TGA GCT TCC |

Ref. seq.: Reference sequence

**Table S6.** RT-PCR primer sequences for genes encoding proteasomal subunits

| **Gene Name** | **Symbol** | **NCBI Ref Seq** | **Direction** | **Primer sequence (5’-3’)** |
| --- | --- | --- | --- | --- |
| Proteasome subunit, alpha type 1 | PSMA1 | NM_002786.1 | Forward | GTT CAG CCA CAG TTG GTC |
|  |  |  | Reverse | GTC CTG TTC TGC AGG AAG |
| Proteasome subunit, alpha type 7 | PSMA7 | NM_002792.3 | Forward | GCA GGA GGC CGT CAA GA |
|  |  |  | Reverse | CTC GCC TCA TGA CAG CAA |
| Proteasome 26S subunit, non-ATPase 1 | PSMD1 | NM_002807.3 | Forward | GCC AGA GCC ACT AAC TGG |
|  |  |  | Reverse | GCC ACA ATA AGC CAT GGC |
| Proteasome activator subunit 1 (PA28 alpha) | PSME1 | NM_006263.3 | Forward | CAC TCC ACT CCT TGT GC |
|  |  |  | Reverse | CAC CTT CTC CTG GAC AG |
| Homo sapiens glyceraldehyde 3-phosphate dehydrogenase | GAPDH | NM_002046 | Forward | GAA GAC TGT GGA TGG CCC |
|  |  |  | Reverse | CCA TGC CAG TGA GCT TCC |

Ref. seq.: Reference sequence

**Table S7.** Quantitative real-time PCR and RT-PCR primer sequences for polyamine-metabolism related genes

| **Gene Name** | **Symbol** | **NCBI Ref Seq** | **Direction** | **Primer sequence (5’-3’)** |
| --- | --- | --- | --- | --- |
| Homo sapiens ornithine decarboxylase 1 | ODC1 | NM_002539 | Forward | GAG CCC GGC AGA TAC TAT GT |
|  |  |  | Reverse | GCT TTA CAT GTG CGT GGT CA |
| Homo sapiens spermidine synthase 1 | SRM1 | NM_003132 | Forward | CGA AAG GTG CTG ATC ATC GG |
|  |  |  | Reverse | CAA AAC CGT CAC CCA CAT GT |
| Homo sapiens spermidine/spermine acetyltransferase 1 | SAT1 | NM_002970 | Forward | ACC TAT GAC CCG TGG ATT GG |
|  |  |  | Reverse | TGC TAC CAA GAA GTG CAT GC |
| Homo sapiens polyamine oxidase | PAOX | NM_152911 | Forward | CAA GAA GGA GAT TGG CCA GC |
|  |  |  | Reverse | CAG CAC GGT ATA CTC CCC AA |
| Homo sapiens glyceraldehyde 3-phosphate dehydrogenase | GAPDH | NM_002046 | Forward | GAA GAC TGT GGA TGG CCC |
|  |  |  | Reverse | CCA TGC CAG TGA GCT TCC |

Ref. seq.: Reference sequence

**Figures**

**Supplementary Figure S1.**

**Supplementary Figure S1.** **Clustering of gene expression in MNPs@SiO_2_(RITC)-treated HEK293 cells compared with untreated controls.** Forty eight genes related to UPS were differentially expressed by > 1.25-fold in 1.0 μg/μl MNPs@SiO_2_(RITC)-treated cells. MultiExperiment Viewer (MeV) diagram showing the correlation of expression difference between MNPs@SiO_2_(RITC)-treated and untreated cells. Genes in boxes showed the same tendency of up- and down-regulation in gene expression with the two concentrations of MNPs@SiO_2_(RITC). Red and green indicate up- and down-regulated genes, respectively.

**Supplementary Figure S2.**

**Supplementary Figure S2. RT-PCR analysis of selected proteasomal subunit genes.** Semiquantitative analysis of ubiquitin proteasome pathway-related genes using RT-PCR. HEK293 cells were treated with 0.1 and 1.0 μg/μl of silica NPs for 12 h. RT-PCR was performed using specific primers for target genes PSMA1, PSMA7, PSMD1, and PSME1. Gene expression levels of the target genes were normalized relative to the corresponding means in non-treated controls. Data show means ± S.D. of three experiments repeated independently.

**\**

**Supplementary Figure S3.**

**Supplementary Figure S3.** **Formation of inclusion bodies in cells treated with MNPs@SiO_2_(RITC).** Characterization of ubiquitin and MNPs@SiO_2_(RITC)-positive inclusion bodies in Synph-293 cells treated with lactacystin (1 μM). Cells were treated with 0.1 and 1.0 μg/μl of MNPs@SiO_2_(RITC) for 48 h followed by immunostaining. The proteasome inhibitor lactacystin was added to the cells at 36 h. Green, ubiquitin; red, MNPs@SiO_2_(RITC); blue, DAPI. Scale bar = 10 μm.

**Supplementary Figure S4.**

**Supplementary Figure S4. Evaluation of cytotoxicity and ROS generation in MNPs@SiO_2_(RITC)-treated neuronal cells. a,** MTS assay of MNPs@SiO_2_(RITC)-treated SH-SY5Y cells and primary cortical and dopaminergic neurons. Cells were treated with 0.1 μg/μl and 1.0 μg/μl MNPs@SiO_2_(RITC) for 12 h, followed by cell viability assessment using MTS assay. **b,** ROS generation in MNPs@SiO_2_(RITC)-treated SH-SY5Y cells and primary cortical and dopaminergic neurons. To evaluate ROS generation, cells were treated with 0.1 μg/μl and 1.0 μg/μl of MNPs@SiO_2_(RITC) for 12 h, followed by fluorescence measurement after staining with DCFH-DA. Intensities were normalized relative to controls. ROS generated by MNPs@SiO_2_(RITC)-treated cells were compared with normalized controls. **p* value < 0.05 in one-way ANOVA compared to control were significantly different. Data represent mean values ± S.D. related to control of three independent experiments.

**Supplementary Figure S5.**

**
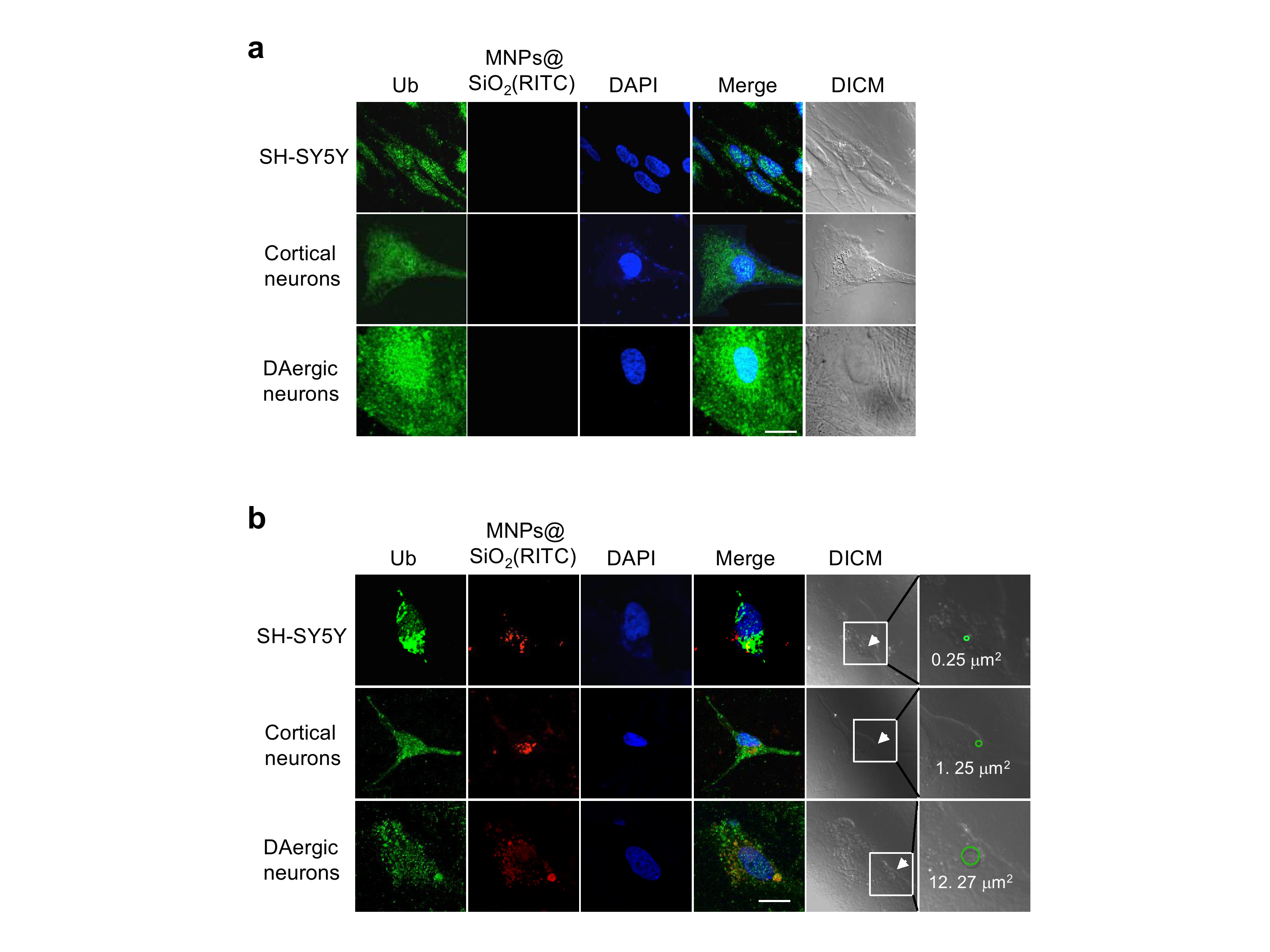
**

**Supplementary Figure S5.** **Characterization of ubiquitin and MNPs@SiO_2_(RITC)-positive cytoplasmic inclusions in SH-SY5Y cells and primary cortical and dopaminergic neurons.** **a,** Untreated cells did not show formation of inclusion bodies. **b,** Cells were treated with 0.1 μg/μl of MNPs@SiO_2_(RITC) for 48 h and subjected to immunostaining. Green, ubiquitin; red, MNPs@SiO_2_(RITC); blue, DAPI. DAergic neurons, dopaminergic neurons. Scale bar = 10 μm.

**Supplementary Figure S6.**

**
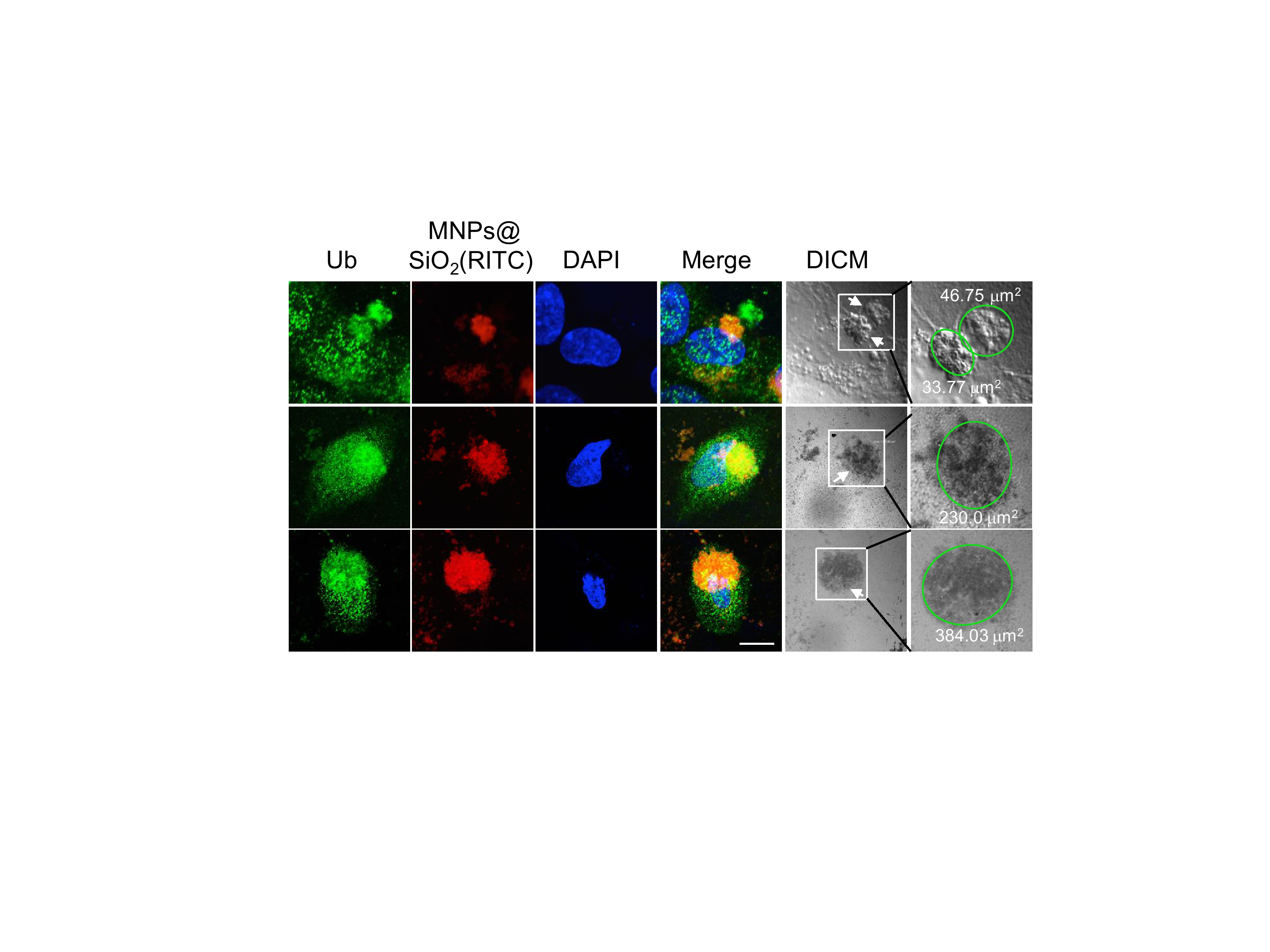
**

**Supplementary Figure S6. Characterization of ubiquitin and MNPs@SiO_2_(RITC)-positive irregular cytoplasmic inclusions in primary dopaminergic neurons.** Cells were treated with 1.0 μg/μl of MNPs@SiO_2_(RITC) for 48 h followed by immunostaining. Green, ubiquitin; red, MNPs@SiO_2_(RITC); blue, DAPI. Scale bar = 10 μm.

**Supplementary Figure S7.**

**Supplementary Figure S7. Characterization of primary cortical and dopaminergic neurons.** Rat primary cortical and dopaminergic neurons were treated with 0.1 μg/μl of MNPs@SiO_2_(RITC) followed by immunostaining. **a,** Immunofluorescence of cortical neurons stained with anti-NeuN antibody. **b,** Immunofluorescence of dopaminergic neurons stained with anti-TH antibody. Scale bar = 10 μm.

**Supplementary Figure S8.**

**Supplementary Figure S8. Synphilin-1 co-localization with MNPs@SiO_2_(RITC) in Synph-293 cells treated with MNPs@SiO_2_(RITC).** Synph-293 cells were treated with 0.1 and 1.0 μg/μl of MNPs@SiO_2_(RITC) for 48 h, followed by immunostaining with FLAG antibody for detection of synphilin-1. Untreated control cells did not form inclusion bodies (upper panel). Small inclusion bodies were formed in the MNPs@SiO_2_(RITC)-treated cells (middle and lower panels). Synph-1, synphilin-1; green, synphilin-1; red, MNPs@SiO_2_(RITC); blue, DAPI.
